# Influence of Constant Magnetic Field upon Fatigue Life of Commercially Pure Titanium

**DOI:** 10.3390/ma15196926

**Published:** 2022-10-06

**Authors:** Krestina Aksenova, Dmitrii Zaguliaev, Sergey Konovalov, Vitalii Shlyarov, Yurii Ivanov

**Affiliations:** 1 Department of Natural Sciences, Siberian State Industrial University, 654007 Novokuznetsk, Russia; 2 Plasma Emission Electronics Laboratory, Institute of High Current Electronics of the Siberian Branch of the Russian Academy of Sciences, 634055 Tomsk, Russia

**Keywords:** VT1-0 titanium, magnetic field, multicycle fatigue, fracture surface, crystalline structure, phase composition, physical properties

## Abstract

Cyclic tests of the multicycle fatigue of commercially pure titanium were performed under normal conditions (without a magnetic field) and after exposure to a constant magnetic field of varying density (B = 0.3, 0.4, 0.5 T). It was shown that the application of the constant magnetic field of varying density led to a fold increase in the average number of cycles to destruction of the VT1-0 titanium samples by 64, 123, and 163%, respectively. Scanning electron microscopy revealed that the magnetic field led to a 1.45-fold increase in the critical length of the fracture (the width of the fatigue crack growth zone) and a 1.6-fold decrease in the distance between the fatigue striations in the accelerated crack growth zone of the destroyed titanium samples. It was established that a subgrain (fragmented) structure formed in the area of the fatigue growth of the fracture of the titanium samples. The size of the subgrains corresponded to the spaces between the fatigue striations, which had an inhibitory influence on the microcrack propagation. Collectively, the revealed facts are indicative of a higher material resistance to fatigue fracture propagation and increased operation resources under the fatigue tests in the magnetic field, which correlates with the data on the growth of the average number of cycles to fracture of the VT1-0 titanium samples.

## 1. Introduction

External energy deposition is widely used to increase the service life of metal elements and constructions, which are mainly damaged by fatigue together with corrosion and wear [1,2]. The traditional methods of external energy deposition, such as thermal, thermochemical, electron beam, plasma, laser treatment, and others, influence the structure, physical, and mechanical properties of metals, including their fatigue resistance, due to the rearrangement of the dislocation substructure and phase composition of metals. For example, the experimental results concerning the effect of electric discharge machining on the fatigue crack growth in aluminum alloys obtained by the authors of [3,4] allowed the conclusion that an increase in the fatigue life is associated with the effect of crack shielding due to the localized melting caused by a pulse electric current of 90 A/mm^2^. In [5,6], it was established that after the laser hardening of Ti–6Al–4V alloy, the cycle life increases by 2.4–42.7% depending on the number of laser passes and reaches its maximum (33,200 cycles) after the 3-time pass. In [7], laser shock processing of Ti-17 alloy led to the growth of its fatigue crack resistance from 1.68 × 10^5^ to 4.05 × 10^5^ and 2.90 × 10^5^ cycles after being treated with laser energy of 20 and 30 J, respectively.

There are other equally effective but less studied methods of external energy deposition, such as magnetic fields. As an effective action parameter, the magnetic field is extremely useful for modification of the microstructure and optimization of the material properties [8,9,10,11,12,13,14,15]. The application of the magnetic field in treating the surface of hypereutectic silumin results in a refinement of the primary silicon, which improves its hardness and microhardness [9]. In Mg-Al-Gd alloy subjected to a constant magnetic field of 1 T applied during homogenization under 525 °C, microstructure changes and mechanical properties improvement were observed [12].

The results of the mechanical tests of titanium alloy TS4 [16] showed that after being treated with an intense pulsed magnetic field of 3 T, the material strength increased by 7.6%, which is explained by the strengthening of the dislocations according to the authors. In the course of tensile tests of the titanium alloy, it was found that material subjected to an intense constant magnetic field became more ductile and its average elongation was 12–13%, which is 31.7% larger in comparison to that of the untreated sample, which was 10.01% [17]. Treatment with constant magnetic fields also improves TS4 alloy strengthening: its microhardness increased by 8.09% when the alloy was subjected to 2 T induction [18]. For VT3-1 alloy, the application of constant and pulsed magnetic fields of varying intensity increases its plasticity [19,20].

Extensive studies were also conducted concerning the influence of the magnetic fields upon the fatigue destruction of various metals and alloys [21,22,23,24].

Kyoung et al. [22] presented example studies of AISI 8620 steel samples, which were exposed to a magnetic field of 10 T for 0, 10, and 20 days. This exposure led to an increase in fatigue life due to the increase in the yield strength and resistance to rupture and a reduction in the elastic modulus. The authors in [23] also observed an increase in the fatigue life of 35CrMo steel exposed to a magnetic field of 1.2–1.3 T of 10–15%. The authors of [24] associated the significant (more than three-fold) increase in the fatigue life of EN8 steel and AA2014-T6 alloy treated by a variable magnetic field of 0.54 T with the growth of residual compressive stresses in the near-surface layers of both metals as a result of the treatment. The problems of the influence of the external magnetic field on the fatigue properties of the materials being welded were studied in [25,26,27,28]. Thus, [28] showed that the application of a magnetic field of 3 mT in the process of welding of 2205 steel allowed an increase in its resistance to fatigue crack nucleation and fracture toughness. The authors attributed this change to the evolution of the microstructure during the thermal cycle included in the welding process.

The conclusion can be made that the nature of the impact of constant and pulsed magnetic fields on the structure and deformation properties, especially on the fatigue resistance, has been understudied. As titanium is widely used in the aerospace industry, where the majority of metal failures are caused by fatigue breakdown and the magnetic field is an environmental (space) factor, the current paper is highly relevant for understanding the physical nature of the effect of magnetic fields of varied intensity on the fatigue behavior of titanium. Earlier, our research team conducted research on the influence of the constant magnetic fields on the VT1-0 titanium microhardness and creep rate [29,30,31]. In the present paper, the authors completed an experimental study on the influence of a constant magnetic field with a density ranging from 0.3 to 0.5 T (in situ) on the fatigue life of commercially pure VT1-0 titanium subjected to cyclic asymmetric loads.

## 2. Materials and Methods

**Figure 1:**
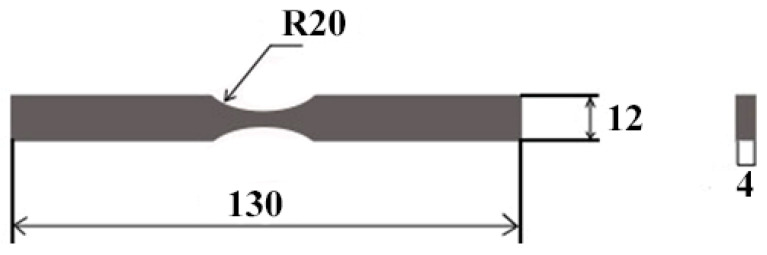
Dimensions of the VT1-0 titanium sample used for the fatigue tests. (unit: mm).

The material used in this study was commercially pure titanium VT1-0 similar to Grade 2 according to American standard ASTM B348 [32], characterized by the following chemical composition: 99.24–99.7% Ti, Fe < 0.25%, C < 0.07%, Si < 0.1%, N < 0.04%, O < 0.2%, H < 0.01%. Alloy VT1-0 has the following mechanical properties at room temperature: tensile strength (σ_b_ = 375 MPa), yield strength (σ_0.2_ = 295–410 MPa), relative elongation (δ_5_ = 20–30%), Brinell hardness (HB = 13.1–16.3 MPa), and Poisson’s ratio (υ = 0.32). The titanium samples under study had the form of a rectangular block with a size of 4 × 12 × 130 mm according to GOST 25.502-79 (Figure 1). The fracture (stress concentrator) was imitated by two cuts in the form of a semicircle with a 20 mm radius in the central part of the sample. The samples were mechanically ground by sandpaper with a reducing fineness of abrasive particles. After the sandpaper grinding, the samples were polished with diamond paste with abrasive particles up to 1 µm in size (until a mirror finish). The surface of the sample in the region of the stress concentrator was prepared to reduce the surface roughness as it is known that under cyclic loads, part destruction is associated with the development of fatigue cracks immediately in the surface layer [1].

**Figure 2:**
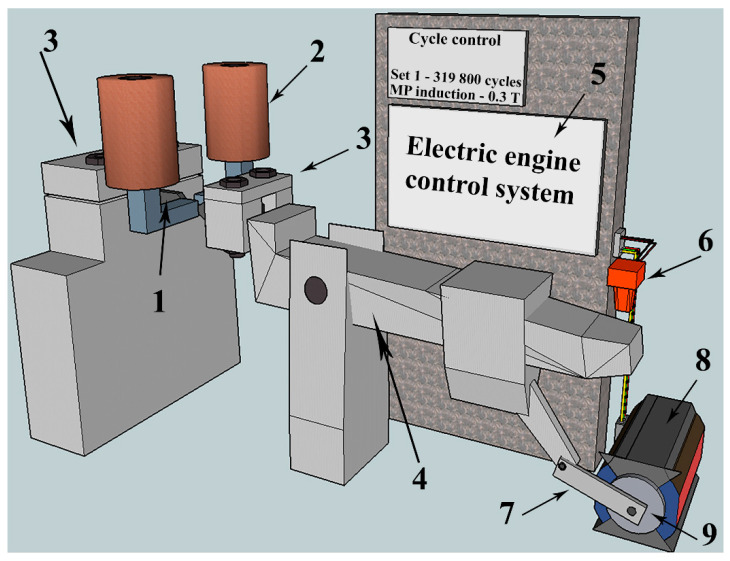
The unit used for the fatigue tests in a constant magnetic field. 1—sample, 2—electromagnet, 3—the unit for fixing the sample, 4—leverage mechanism, 5—control system, including the unit controlling the motor speed and the cycle counter, 6—photo censor, 7—eccentric, 8—motor, 9—axle.

The tests for high-cycle fatigue were completed on a special unit developed by the team of authors in the form of cyclic asymmetric cantilevered bending, the image and layout of which are shown in Figure 2. Sample (1) is fixed with clamps (3). One end of the sample is motionless and the other is subjected to the fatigue load. The bending is carried out with the leverage mechanism (4), which is connected to the axle (9) rotated by the electric motor (8). The stress amplitude can be changed with the eccentric part (7). The photo censor (6) is used to register the number of loading cycles. The motor speed can be changed by altering the voltage supplied to the electric motor winding (8). The magnetic field source (2) is placed inside the rigid frame, and the field density is changed by regulation of the electric current flowing through the inductors.

The number of cycles withstood by the samples before destruction was determined during the tests. All the tests were conducted at room temperature (~300 K). The frequency of sample loading by bending was ~3.3 Hz. The upper value of the load cycle stress and loading amplitude were experimentally selected so that the sample could withstand at least 10^5^ loading cycles before failure. Based on the results of trial tests, for further multicycle fatigue testing of titanium samples in the magnetic field and without it, a mode was chosen in which the loading amplitude was a = 3.5 mm, the maximum stress of the load cycle σ = 33 MPa, and the cycle asymmetry coefficient R = 0.1. The fatigue tests of the samples were conducted for the samples in their initial condition (without the application of the magnetic field) and in the magnetic field (in situ) with density B = 0.3, 0.4, and 0.5 T. The value of the magnetic field density was controlled with the help of militeslameter TPU with an accuracy of up to 0.01 mT. At least 10 samples were tested for each value of magnetic field density.

The scanning electron microscopy (microscope LEO EVO 50) was used to carry out the studies of the fracture surface. The specialized software package for image processing and analysis ImageJ was applied to establish the size of the structural elements. The math software package Origin Pro 8.5 was used to analyze the statistical assessment of the obtained test results’ validity.

## 3. Results and Discussion

As a result of these studies, it was established that commercially pure titanium VT1-0 subjected to multicycle fatigue tests without a magnetic field is destroyed after an average of 121,478 ± 7112 cycles after being exposed to an asymmetric load with a frequency of ~3.3 cycles/s. After the constant magnetic field was applied in the process of testing, the average value of the fatigue life increased significantly. The average number of cycles to fracture under the action of the constant magnetic field was 199,105 ± 15,023 under B = 0.3 T, 270,492 ± 20,505 under B = 0.4 T, and 319,828 ± 27,321 under B = 0.5 T.

**Figure 3:**
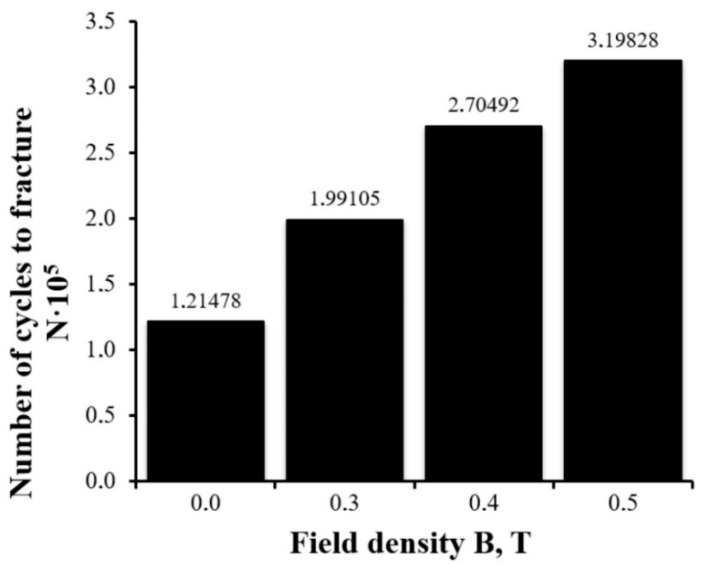
Dependence of the average number of cycles to fracture N upon the value of the field density B under the cyclic fatigue life tests of commercially pure titanium VT1-0.

The dependence of the average number of cycles to fracture N upon the parameters of the external magnetic field (density B) is presented in Figure 3. Analysis of the dependence presented allows us to say that the application of the constant magnetic field with a density of 0.3, 0.4, and 0.5 T resulted in a fold increase in the fatigue life of titanium VT1-0 samples of 64, 123, and 163%, respectively.

It is obvious that the fold increase (more than by 2.5 times) in the number of cycles to fracture indicates the condition of the fracture surface of the titanium samples. To study the fracture surface, we chose the titanium samples for which the numbers of cycles to fracture were closest to the average: 121,558 in the initial state, 191,320 under B = 0.3 T, 269,012 under B = 0.4 T, and 329,151 under B = 0.5 T.

**Figure 4:**
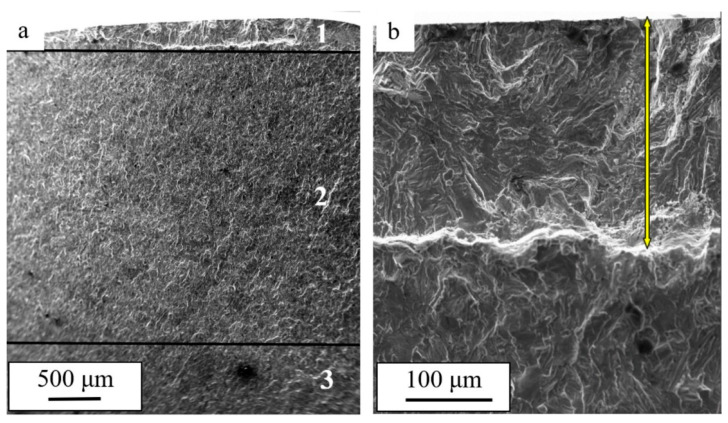
The structure of the fracture surface of a titanium sample subjected to fatigue tests in a magnetic field under B = 0.4 T. In (**a**), the figures indicate the characteristic areas of fatigue destruction: 1—the area of fatigue growth of the fracture; 2—the area of accelerated growth of the fracture; 3—the rupture area. The arrow in (**b**) indicates the area of fatigue growth of the fracture.

It is known that the process of fatigue fracture develops locally with time and the destruction of the whole sample occurs after a certain critical state is achieved [1]. In general, the fracture surface, independently of the test conditions, has three specific areas (Figure 4a): the area of fatigue growth of the fracture (1) (Figure 4b, indicated with an arrow); the area of accelerated growth of the fracture (2); and the rupture area (3) [33]. The deformation processes that take place under the fatigue tests most intensively develop in the area of fatigue growth of the fracture and much less intensively in the rupture area.

**Figure 5:**
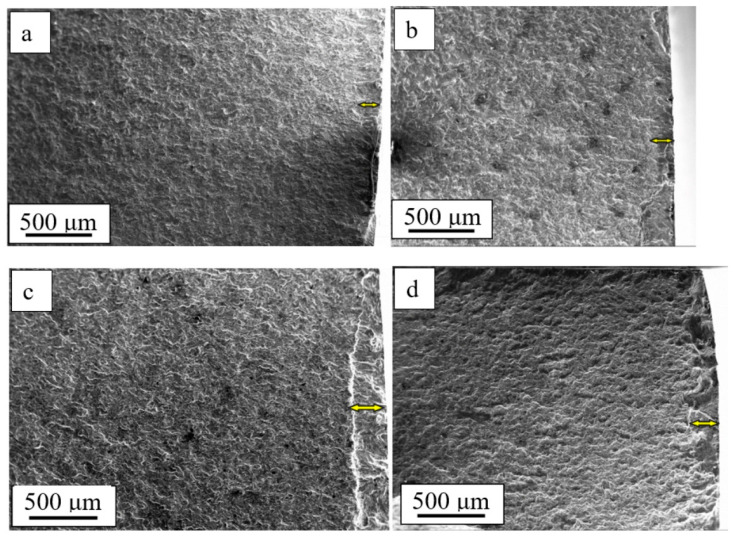
Structure of the titanium fracture surface subjected to fatigue tests in the magnetic field: (**a**) 0 T; (**b**) 0.3 T; (**c**) 0.4 T; (**d**) 0.5 T. The arrow indicates the zone of fatigue crack growth.

Figure 5 shows images of the titanium samples’ surface that fractured in the magnetic field of different magnitudes. In the course of the study of the fracture surface of the titanium VT1-0 samples, it was established that the width of the area of fatigue growth of the fracture depends on the field density and reached its maximum values (h = 264 µm) under B = 0.4 T (Figure 5c). Under the fatigue tests without the magnetic field, h = 182 µm (Figure 5a). The width of the area of fatigue growth of the fracture is equated to the critical length of the fracture [34]. Therefore, under the fatigue loading in the magnetic field, the critical length of the fracture increased by 1.45 times, which indicates an increase in the operation resources of the material.

**Figure 6:**
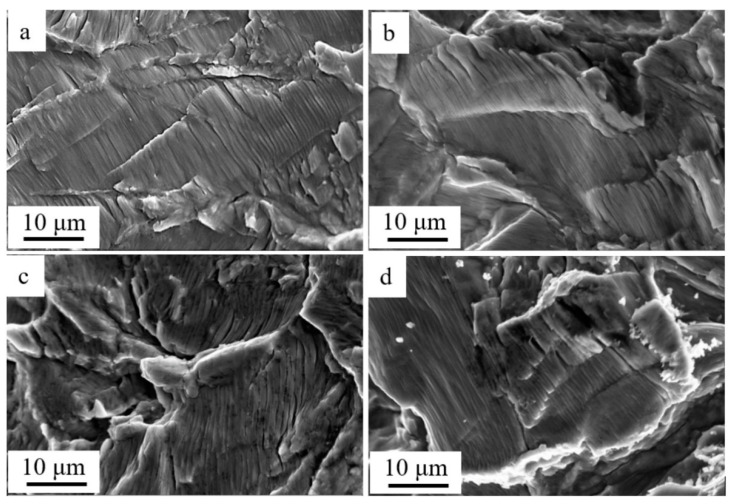
Fatigue striations formed in titanium as a result of fatigue failure in the constant magnetic field: (**a**) 0 T; (**b**) 0.3 T; (**c**) 0.4 T; (**d**) 0.5 T.

Fatigue striations, a typical view of which is shown in Figure 6, are an important mark of cyclic fatigue tests of a material [33,34,35,36]. The term “fatigue striations” denotes successive depressions and peaks or stripes, with slip bands limited by these depressions running parallel to the crack front. With each stress cycle, the crack (fracture) moves forward a certain distance, leaving a line of successive progression marks on the fracture surface. Therefore, these marks represent a trace of the fracture, which, in general, propagates by one step in each stress cycle. The striations are continuous and regular (typical for aluminum alloys) with a decreasing distance between them as the stress levels and propagation rate of the fracture decrease. They are discontinuous and irregular, which is typical for the fracture surface of steels.

With the other conditions associated with the layout of the fatigue stress experiment being equal, the distance between the striations is determined by the ability of the material to resist fatigue fracture propagation: the greater the distance between the striations, the larger the material resistance to cracking and, consequently, the smaller the fracture propagation rate [36]. The studies of the area of accelerated growth of the fracture we completed show that the average distance between the fatigue striations in the titanium samples depends on the magnetic field density and decreased from 0.78 µm under B = 0.0 T (Figure 6a) to 0.49 µm under B = 0.5 T (Figure 6d). Consequently, the step of a fracture during one fatigue stress cycle in the magnetic field (under B = 0.5 T) in the titanium sample was 1.6 times smaller in comparison to the sample fractured without the magnetic field. This means that the titanium sample destroyed in the magnetic field is more resistant to the propagation of the fatigue fracture.

**Figure 7:**
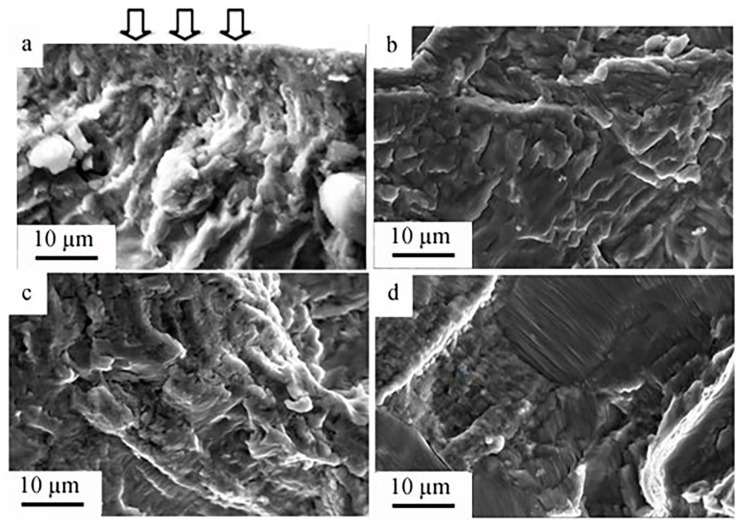
Block (subgrain) structure formed in the surface layer of titanium destroyed during the fatigue tests in the magnetic field: (**a**) 0 T; (**b**) 0.3 T; (**c**) 0.4 T; (**d**) 0.5 T. The arrows in (**a**) indicates the surface of the sample.

Under the conditions of fatigue loading, the process of the hardened surface layer formation is developed [33]. Figure 7 shows images of the surface layer structure of the destroyed titanium samples (the zone of fatigue crack growth). A feature of the zone of fatigue crack growth is the formation of a subgrain (fragmented) structure. The sizes of the subgrains depend on the magnitude of the magnetic field induction and varied from 0.56–0.87 µm for the sample destroyed in the absence of the magnetic field (Figure 7a) to 0.47–0.65 µm for the sample destroyed in the magnetic field at B = 0.5 T (Figure 7d).

The completed studies show that the subgrain structure formation in the area of the fatigue growth of the fracture influences the propagation of the microfracture. Namely, the distance between the fatigue striations corresponds to the size of the subgrains. Analysis of the results presented in Figure 6 and Figure 7 points to the fact that the fatigue striations in the surface layer are spaced at a distance correlating to the size of the subgrains. Consequently, the subgrain boundaries in the fatigue fracture growth area have an inhibitory influence on the propagating microfracture.

The facts revealed in the result of the fracture surface analysis indicate a higher material resistance to the propagation of fatigue crack and an increase in its service life during fatigue tests in the magnetic field. It correlates with the data on an increase in the average number of cycles to fracture of the VT1-0 titanium samples. However, more in-depth structural studies are needed in order to develop the hypothesis of the influence of weak magnetic fields (up to 0.5 T) on the deformation behavior of paramagnetic metallic materials under the simultaneous action of alternating loads and magnetic fields of different strengths. In this regard, layer-by-layer electron microscopic studies of the fine structure, phase composition, and dislocation substructure depending on the distance to the fracture surface (0, 150, 300, and 600 μm) of commercially pure titanium VT1-0, which will be destroyed during multicycle fatigue tests without a magnetic field and in a constant magnetic field of 0.3 and 0.5 T, are planned.

## 4. Conclusions

Multicycle fatigue tests of VT1-0 titanium samples were carried out under the action of a constant magnetic field with a various density and without it. It was shown that, with the increase in the field density, the average number of cycles to fracture of the titanium samples increased from N = 121,478,121,478 ± 7112 (B = 0 T) to N = 319,828 ± 27,321 (B = 0.5 T). Therefore, exposure to the constant magnetic field with a density of 0.3, 0.4, and 0.5 T resulted in a fold increase in the fatigue life of VT1-0 titanium of 64, 123, and 163%, respectively.

Fractographic analysis of the fracture surface of commercially pure titanium subjected to multicycle loading allowed the conclusion that, regardless of the conditions of the fatigue tests, the structure of the fractured samples has three areas: the area of fatigue growth of the fracture, the area of accelerated growth of the fracture, and the rupture area. It was established that the width of the area of the fatigue growth of the fracture depends on the magnetic field density and showed maximum values (h = 264 µm) under B = 0.4 T. Under the fatigue tests without the magnetic field, h = 182 µm, which is indicative of an increase in the critical length of the fracture (width of the area of the fatigue growth of the fracture) of 1.45 times. It was shown that the average distance between the fatigue striations in the titanium samples also depends on the magnetic field density and decreased from 0.78 µm under B = 0.0 T to 0.49 µm under B = 0.5 T. Consequently, the critical length of the fracture in the titanium sample destroyed in the magnetic field (under B = 0.5 T) was 1.6 times smaller than that in the sample destroyed without the field.

The formation of a subgrain (fragmented) structure is a specific feature of the area of fatigue growth of the fracture in titanium VT1-0. The sizes of the subgrains of the sample destroyed without being exposed to the magnetic field were 0.56–0.87 µm while those of the sample destroyed under the cyclic tests in the magnetic field under B = 0.4 T were 0.67–1.1 µm. Analysis of the obtained results showed that the fatigue striations in the surface layer were spaced at a distance correlating to the size of the subgrains. Consequently, the subgrain boundaries in the area of fatigue growth of the fracture had an inhibitory influence on the propagating microfracture.

Therefore, the revealed facts suggest a higher resistance to fatigue fracture propagation and an increase in the operation resources of titanium VT1-0 under the fatigue tests in the constant magnetic field.

## Data Availability

Not applicable.
